# Effect of Different Dietary Patterns on Patients with Depressive Disorders: A Systematic Review and Meta-Analysis of Randomized Controlled Trials

**DOI:** 10.3390/nu17030563

**Published:** 2025-01-31

**Authors:** Rahele Tavakoly, Alina Moosburner, Dennis Anheyer, Holger Cramer

**Affiliations:** 1 Institute of General Practice and Interprofessional Care, University Hospital Tübingen, 72076 Tübingen, Germany; alina.moosburner@med.uni-tuebingen.de (A.M.); dennis.anheyer@uni-wh.de (D.A.); 2 Robert Bosch Center for Integrative Medicine and Health, Bosch Health Campus, 70376 Stuttgart, Germany; 3 Department of Psychology and Psychotherapy, University of Witten/Herdecke, 58455 Witten, Germany

**Keywords:** dietary patterns, diet, depressive disorder, clinical trials, systematic reviews, meta-analysis

## Abstract

**Objectives**: This study aimed to investigate the effects of different dietary patterns on depressive disorders. **Methods**: PubMed/MEDLINE, Cochrane CENTRAL, Embase, PsycINFO, Scopus, and ProQuest databases were systematically searched until 30 April 2024 for randomized controlled trials (RCTs) assessing the effects of different dietary patterns on depressive symptoms in adults with depressive disorders. Secondary outcomes included remission rates, quality of life, and safety. Standardized mean difference (SMD) and 95% confidence intervals (CIs) were aggregated using a random-effects model. Study quality was assessed with the Cochrane Risk of Bias (RoB)-2 tool, and certainty of evidence was determined using the Grading of Recommendations, Assessment, Development, and Evaluation (GRADE) approach. **Results**: Five RCTs (*n* = 952) were included, all investigating the Mediterranean diet (MD) in individuals with major depressive disorder or elevated depression levels. The analysis found no significant effect of short-term MD intervention on depression severity compared to active (SMD = −1.25 [95% CI: −5.11 to 2.61]) or passive (SMD = −0.22 [95% CI: −0.74 to 0.29]) controls. There was no effect on quality of life compared to active controls (SMD = 0.71 [95% CI = −3.38 to 4.79]). Intermediate and long-term results were similar. The overall risk of bias was “some concerns”, and the certainty of evidence was “very low” for most of the results. **Conclusions**: The MD appears to have no potential influence on major depressive disorder. However, this finding should be interpreted cautiously due to the limited number of RCTs. Further studies on dietary patterns and depressive disorders are needed for more robust conclusions. **Systematic Review Registration**: PROSPERO registration no. CRD42024541885.

## 1. Introduction

Psychological disorders are now recognized as the seventh leading contributor to the global burden of disease. Among these, depressive disorders are the primary cause of disability in individuals over the age of 14, affecting 279.6 million people worldwide, with a lifetime prevalence of 3.4% to 4.2% [1]. Depressive disorders are associated with unemployment, poor physical health, low quality of life, and impaired social functioning [2]. This condition places a considerable burden not only on individuals but also on society, with an estimated annual economic cost of USD 1 trillion due to lost productivity and increased demand for healthcare services from anxiety and depressive disorders [3].

Standard treatments for depressive disorders comprise pharmacological and psychological interventions, both of which have demonstrated efficacy [4,5]. However, these approaches alone do not lead to sustained symptom remission for all individuals [6,7].

In this context, lifestyle-based interventions, including nutrition, have emerged as complementary strategies in the treatment of depressive disorders. Earlier studies investigating the effect of nutrition on depressive disorders have primarily focused on isolated nutrients, such as selenium [8], Omega-3 fatty acids [9], or vitamin D [10]; isolated foods like nuts [11] or fish [12]; or bioactive compounds like polyphenols [13] or caffeine [14]. However, nutrients, foods, and bioactive compounds are consumed in combination, and no single nutrient or food can fully explain the therapeutics effects of a nutrition approach [15].

The dietary pattern approach, as a newer direction in nutritional epidemiology, considers overall dietary intake as well as the interactions and synergistic effects between various nutrients and foods [16]. A dietary pattern is defined as “the quantities, proportions, variety, or combination of different foods and drinks in diets, and the frequency with which they are habitually consumed” according to the Dietary Guidelines Advisory Committee 2020 [17]. Several dietary patterns have been recognized, with an increasing number emerging, including Atkins, ketogenic, Nordic, paleolithic, Mediterranean, vegetarian, macrobiotic, vegan, or Dietary Approaches to Stop Hypertension (DASH), among the most common.

Previous systematic reviews of observational studies have explored the relationship between various dietary patterns and depressive symptoms. A recent study in older adults showed that adherence to the Mediterranean diet may counteract the development of depression and alleviate depressive symptoms, likely due to the anti-inflammatory properties of this diet [18]. Current literature suggests a link between the inflammatory potential of a diet and the risk of depression, in contrast to an anti-inflammatory diet [19]. Another systematic review of adults with depression found no significant association between a vegetarian diet and depression [20]. However, there is some evidence suggesting that diets rich in antioxidants, characterized by high total dietary antioxidant capacity (dTAC) scores, may be inversely associated with depression [21].

There are also several systematic reviews of interventional studies examining the effects of various dietary patterns on depressive status in different populations. In one systematic review, which included various types of studies, the RCTs revealed no association between vegetarian and vegan diets and depression [22]. In another systematic review of RCTs involving both healthy and patient population, almost 19% of studies indicated beneficial effects of ketogenic diets on mental health and depression [23]. Additionally, in a review of different study types, most RCTs provided evidence supporting the beneficial effects of the DASH diet on mental health [24]. However, the number of systematic reviews on interventional studies in patients diagnosed with clinical depressive disorders is limited, and they have concentrated on a single dietary pattern. One such review indicated that Mediterranean dietary (MD) interventions can reduce the severity of depressive symptoms in adults with major depression [25].

Therefore, the aim of this study was to investigate the effects of different dietary patterns on depressive symptom severity in adults with depressive disorders. Secondary outcomes included remission rates, health-related quality of life, and safety. To the best of our knowledge, this study is the first comprehensive assessment of the effects of dietary patterns in depressive disorders based on randomized controlled trials (RCTs).

## 2. Materials and Methods

This systematic review was performed following the guidelines outlined in the Cochrane Collaboration Handbook for Systematic Reviews of Interventions [26] and reported in accordance with the Preferred Reporting Items for Systematic Reviews and Meta-Analyses (PRISMA) 2020 statement [27]. The study protocol was prospectively registered in PROSPERO (CRD42024541885). One researcher (R.T.) performed the literature search, while two researchers (R.T. and A.M.) independently carried out screening, trial selection, data extraction, risk of bias assessment, and certainty of evidence evaluation. Any disagreements were resolved by a third researcher (H.C. for literature search, screening, and trial selection; D.A. for data extraction, risk of bias assessment, and certainty assessment) if the discussion between the two researchers did not result in an agreement. Study authors were contacted for additional information when necessary.

### 2.1. Eligibility Criteria

The eligibility criteria were based on the PICOS (population, intervention, comparison, outcome, and study design) framework [26]. To be included, studies retrieved from the literature search had to meet the following. Full details of the eligibility criteria are outlined in Table 1.

- Population:

Patients with a clinical diagnosis of any type of depressive disorders according to the Diagnostic and Statistical Manual of Mental Disorders (DSM)-V [28] and the International Classification of Diseases (ICD)-11 [29] were included in the review. This encompassed conditions such as disruptive mood dysregulation disorder, major depressive disorder, persistent depressive disorder (formerly known as dysthymic disorder), premenstrual dysphoric disorder, other specified depressive disorder, unspecified depressive disorder, and unspecified mood disorder. Additionally, individuals with elevated levels of depression, as indicated by standard questionnaires, were also included.

- Intervention:

Dietary patterns were defined based on the Dietary Guidelines Advisory Committee 2020 [17], and the names of various dietary patterns were sourced from this reference [17] as well as through additional literature searches to complete the list. The following dietary patterns were identified: South Beach, Atkins, ketogenic, gluten free, prudent, Nordic, paleolithic, plant based, Mediterranean, vegetarian, macrobiotic, vegan, DASH, immunonutrition, fermentable oligosaccharides disaccharides monosaccharides and polyols (FODMAP), low sodium, pescetarian, high fiber, whole grain, traditional, polyphenol, anti-oxidant, anti-inflammatory, fruit and vegetable, flexitarian, and fruitarian.

- Comparison:

Any type of control group, including both active and passive conditions, was considered.

- Outcome:

Primary outcome was change in depressive symptoms severity, and secondary outcomes were changes in remission rate, health-related quality of life, and safety.

- Study design:

RCTs (parallel, crossover, cluster).

### 2.2. Search Strategy

Searches were conducted using the following electronic databases: PubMed/MEDLINE, Cochrane CENTRAL, Embase, PsycINFO, and ProQuest. These databases were searched from their inception to 30 April 2024 without any language restrictions. Additional searches were performed for conference papers in Scopus, key journals in the field, and trial registry data portals, including World Health Organization (WHO) portals, International Standard Randomized Controlled Trial Number (ISRCTN.com), and ClinicalTrials.gov. Finally, the reference lists of all included trials, as well as relevant reviews and systematic reviews, were manually searched. The search terms included “depressive disorders”, “dietary patterns”, and “RCT”. The complete search strategy for each database is provided in Supplementary File Table S1.

### 2.3. Study Selection 

All identified studies were imported into the Mendeley reference manager (Version 2.116.1; Mendeley: Elsevier, Amsterdam, Netherlands), and duplicates were removed. A two-step screening process was applied based on the eligibility criteria. The first step involved screening titles and abstracts, followed by a full-text review of the selected studies.

### 2.4. Data Extraction and Management

#### 2.4.1. Data Extraction

The following data were extracted from the included studies.

- General characteristics: characteristics of the study, participants, interventions, control groups, and outcomes.
- Quantitative data: sample sizes, means, and standard deviations of outcomes for each group to support meta-analysis.
- Safety assessments: reports of adverse events and serious adverse events.

#### 2.4.2. Risk of Bias Assessment

Risk of bias was assessed using the Cochrane Risk of Bias (RoB)-2 Tool for RCTs [30], which evaluates bias across five domains: bias due to the randomization process, bias due to deviations from intended interventions, bias due to missing outcome data, bias in measurement of the outcome, and bias in selection of the reported result. An algorithm was used to generate a proposed item rating for each domain. Judgments were categorized as “low risk”, “some concerns”, or “high risk” of bias for each domain as well as for the overall risk of bias for each RCT.

#### 2.4.3. Measures of Intervention Effect and Data Synthesis

A meta-analysis was performed using R software (version 4.2.3; R Foundation for Statistical Computing, Vienna, Austria;) with the meta package [31] when at least two studies addressed the same outcome.

For continuous outcomes, standardized mean differences (SMDs) with 95% confidence intervals (CIs) were calculated utilizing Hedges’ correction for small sample sizes. Standard deviations were derived from standard errors or confidence intervals when unavailable [26]. A negative SMD for depression severity and a positive SMD for health-related quality of life indicated favorable effects of the dietary pattern intervention compared to the control group. Cohen’s criteria were used to interpret effect sizes: small (SMD = 0.2–0.49), medium (SMD = 0.5–0.79), and large (SMD ≥ 0.8). [32]. For dichotomous outcomes, risk ratios (RRs) with 95% CIs were calculated by dividing the risk of event in the intervention group (number of participants with the outcome divided by the total in the group) by the risk in the control group [26].

Given that considerable between-study heterogeneity was expected, random-effects models were used with the inverse variance approach to evaluate the effects of dietary pattern interventions versus control conditions on continuous outcomes (i.e., between-group differences). Additionally, the Hartung–Knapp small-sample correction was applied to better account for uncertainty when pooling treatment effects from a limited number of heterogeneous studies [33].

Analyses were stratified by follow-up duration: short-term (≤4 months), intermediate-term (4–12 months), and long-term (≥12 months). Additionally, separate analyses were conducted based on the type of control group, distinguishing between active and passive controls.

#### 2.4.4. Heterogeneity

Statistical heterogeneity among studies was assessed using the I² and τ² statistics. I² measures the percentage of heterogeneity in treatment effects, while τ² indicates the underlying heterogeneity between studies and is unaffected by the number of studies or sample size. The interpretation of I² values was as follows: 0–24% (low heterogeneity), 25–49% (moderate), 50–74% (substantial), and 75–100% (considerable) [34]. The restricted maximum-likelihood estimator was used for τ² calculations [26].

#### 2.4.5. Subgroup and Sensitivity Analyses

Subgroup analyses were performed based on depression diagnosis (clinical diagnosis vs. elevated symptoms), age range (≥25 years vs. <25 years), body mass index (BMI) (≥25 kg/m^2^ vs. <25 kg/m^2^), and gender (male vs. female), where data allowed. To assess the robustness of statistically significant results, sensitivity analyses were conducted for studies with a low risk of bias.

#### 2.4.6. Grading the Quality of Evidence

If at least two studies addressed the same outcome, the certainty of evidence for each outcome, based on the follow-up duration and control conditions, was assessed in five domains: risk of bias, indirectness, imprecision, inconsistency, and other considerations (including publication bias, large effect, plausible confounding, and dose response gradient) according to the GRADE (Grading of Recommendations, Assessment, Development and Evaluation) approach [35] and was rated as very low, low, moderate, or high certainty.

#### 2.4.7. Publication Bias

When the meta-analysis included at least ten studies, publication bias was evaluated by visually inspecting funnel plots. Symmetrical funnel plots suggested a low risk of publication bias, while asymmetrical plots indicated a higher risk. Furthermore, publication bias was assessed using a linear regression test (Egger’s test).

#### 2.4.8. Methodological Considerations

In studies with multiple assessments during follow-up [36,37,38,39,40], all data were included and categorized based on the follow-up duration. When a study had more than one assessment within a specific category [36,37,38,40], we selected the assessment closest in timing to those in other studies within the same category. For RCTs reporting outcome measures using multiple scales or tools [39,40,41], we used the measures from the most commonly applied scale or tool within that study.

## 3. Results

### 3.1. Literature Search

**Figure 1:**
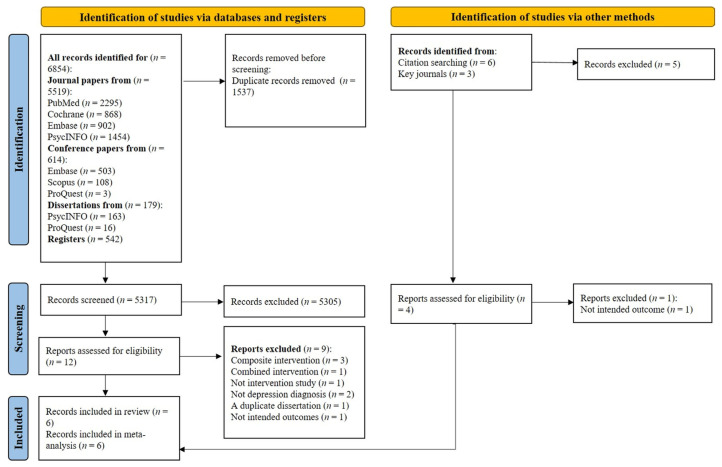
Study flow diagram.

As shown in Figure 1, a total of 5317 papers were initially considered for title and abstract screening after duplicates were removed. Of these, 12 were selected for full-text eligibility assessment, and three were ultimately included in this study. Furthermore, six studies were identified through citation searching and two through key journals. Of these, four were selected for full-text eligibility assessment, resulting in three being included. Two of the included papers [37,38] originated from the same study. One paper focused on health-related quality of life outcomes [38], while the other reported on depressive symptom severity [37].

In total, six papers representing five distinct studies (AMMEND study [36]; PREDI-DEP study [37,38]; SMILES study [41]; MooDFOOD study [39]; and diet, mood, and cognition study [40]) were included in this systematic review and meta-analysis.

Two additional studies assessed psychological distress in participants without specifying depressive disorders or symptoms [42,43]; therefore, these studies were not included in our review.

### 3.2. General Characteristics of the Studies

#### 3.2.1. Study Characteristics

Table 2 summarizes the main characteristics of the included studies. The RCTs were conducted between 2017 and 2023. Three studies were performed in Australia [36,40,41] and three in European counties [37,38,39], with the latter being multicenter studies. Outcome assessors were blinded in all studies except one, which was open-labeled [36]. Furthermore, statistical analyzers were blinded in two trials [39,41]. All RCTs employed a parallel design, except for one that used a factorial design [39]. Recruitment took place in both clinical settings [37,38,41] and community settings [36,39,40,41].

#### 3.2.2. Participant Characteristics

The total baseline sample consisted of 952 participants. Two studies focused on younger adults ages 18–35 years [36,40], while the remaining studies included adults ages 18 and older. The mean participant age ranged from 19.6 years [40] to 51.1 years [37,38], with a median of 40.3 years. Female participants comprised 66.1% of the sample, ranging from 0% [36] to 73.5% [37,38], with a median of 71.6%. Follow-up assessments were completed by 83.5% of participants, with completion rates ranging from 76.8% [39] to 96% [36] (median: 90.7%).

Participants in the RCTs had moderate to severe major depressive disorder diagnosed clinically [36,41]; were in remission from major depressive disorder [37,38]; or exhibited elevated depressive symptoms based on questionnaires, ranging from mild [39] to moderate or higher severity [40]. All RCTs permitted concurrent psychological treatments, such as antidepressants or psychotherapy, except for one trial [39].

Depression diagnoses were performed using several tools, including Beck’s Depression Inventory (BDI) [36,37,38]; Structured Clinical Interview for DSM (SCID) [37,38]; Montgomery–Asberg Depression Rating Scale (MADRS) [41]; Patient Health Questionnaire (PHQ-9); Mini-International Neuropsychiatric Interview (MINI) 5.0 [39]; and Depression, Anxiety, and Stress Scale (DASS-21-D) [40].

#### 3.2.3. Intervention Characteristics

The only dietary pattern assessed in the included studies of our systematic review was the MD. Consequently, no eligible studies on other dietary patterns were available for comparison regarding their effects on depressive disorders.

All included RCTs implemented MD interventions delivered by nutritionists or dietitians. These interventions focused on nutrition education and personalized dietary advice to help participants adopt MD-style eating habits and improve food-related behaviors. Researchers provided supporting materials, such as booklets with information on key Mediterranean foods, seasonal shopping lists, serving sizes, sample meal plans, recipes, dining out tips, strategies for managing time for food preparation, and eating on a budget. Follow-up sessions addressed participants’ questions and clarified doubts [36,37,38,39,40,41]. Furthermore, some trials incorporated food-related behavioral activation approaches in the intervention sessions. These included self-monitoring, functional analysis, promoting positive behaviors, motivational interviewing, goal setting, and discussions on mindful eating [36,39,41].

In some studies, participants were provided with food hampers containing “the main components of MD” [36,41], “olive oil” [37,38], or “olive oil, seeds, nuts, and some spices” [40]. Some trials did not include face-to-face intervention sessions with participants [36,37,38,40]. One study featured both individual and group-based intervention sessions [38]. Another trial used a 2 × 2 factorial design with multi-nutrient supplementation, which included four arms. The two arms relevant to our analysis were the dietary intervention with placebo (intervention group) and the no dietary intervention with placebo (control group) [38].

Intervention duration varied from three weeks [40] to two years [37,38]. Total contact time with nutritionists ranged from 23 min [40] to 810 min [39], with a median of 300 min; however, one study did not report contact time with the nutritionist [37,38]. Adherence to MD interventions was assessed using the Mediterranean Diet Adherence Screener (MEDAS) [36,37,38] or diet compliance score questionnaires developed by the researchers [39,40,41]. One trial also used spectrophotometry to assess compliance with the MD [40]. Most studies [36,39,40,41] showed that intervention groups had improved adherence to the MD compared to control groups.

#### 3.2.4. Control Characteristics

The comparison groups included an active control condition, such as befriending support sessions [36,41], and a passive control condition, which followed participants’ habitual diet [37,38,39,40]. In one study, the control group also received a placebo, as the study utilized a 2 × 2 factorial design, as described earlier [39].

Befriending support sessions followed the same visit schedule and duration as the diet intervention group. During these sessions, participants discussed neutral topics such as movies, news, sports, hobbies, and music or engaged in activities such as cards or board games. Participants in the befriending group were also provided with a gift cart of the same value to the food hampers provided to the intervention group.

#### 3.2.5. Outcome Characteristics

Major depressive disorder was the only condition assessed in the trials included in this systematic review.

The included trials measured several outcomes, including depressive symptom severity [36,37,39,40,41], remission rate [41], health-related quality of life [36,38,39,41] and safety [36,37,38,39]. Depressive symptom severity was measured using the following scales: BDI [36,37], Profile of Mood States (POMS) [40,41], MADRS [41], Hospital Anxiety and Depression Scale (HADS) [41], Clinical Global Impression (CGI-I) [41], PHQ-9 [39], Inventory of Depressive Symptomatology (IDS30-SR) [39], Center for Epidemiologic Studies Depression Scale (CESD-R) [40], and DASS-21-D [40] questionnaires. Remission rate was measured using the MADRS questionnaire [41]. Health-related quality of life was measured using the World Health Organization Quality of Life (WHOQOL-BREF) [36], Short Form Health Survey (SF-36) [38], WHO wellbeing scale 5 [41], and EuroQol instrument (EQ)-5D-5L [39] questionnaires. We used the PHQ for the study by Bot et al. [39], MADRS for the study by Jacka et al. [41], and DASS for the study by Francis et al. [40], as these studies employed multiple tools to measure depressive symptoms. Safety was assessed through side effects or adverse events [36,37,38] and serious adverse events [39].

### 3.3. Risk of Bias Assessment

**Figure 2:**
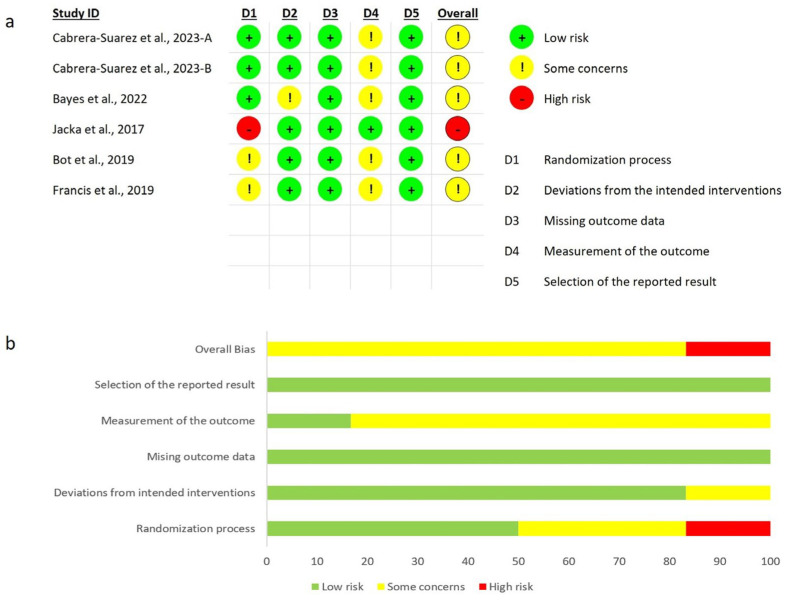
(**a**) Risk of bias assessment for included studies; (**b**) judgements on each domain as a percentage. Cabrera-Suarez et al., 2023-A [38], Cabrera-Suarez et al., 2023-B [37], Bayes et al., 2022 [36], Jacka et al., 2017 [41], Bot et al., 2019 [39], Francis et al., 2019 [40].

The assessed risk of bias for the six included studies is shown in Figure 2. According to the RoB2 tool, the overall risk of bias in all the studies was rated as “some concerns”, except for one study [41], which was rated as “high risk” due to bias in the “randomization process” domain. The domain with the most concerns about bias across the RCTs was the “measurement of the outcome”. Given the nature of the interventions and the self-assessed outcomes, outcome assessors were aware of the intervention received, which could have influenced the assessment of the outcomes.

### 3.4. Analyses of Overall Effects

For the intermediate follow-up category of quality of life, only one measurement was included [39]. Additionally, only one study assessed the remission rate outcome [41], so we did not conduct a meta-analysis for these outcomes.

#### 3.4.1. Effect on Depressive Symptoms Severity

**Figure 3:**
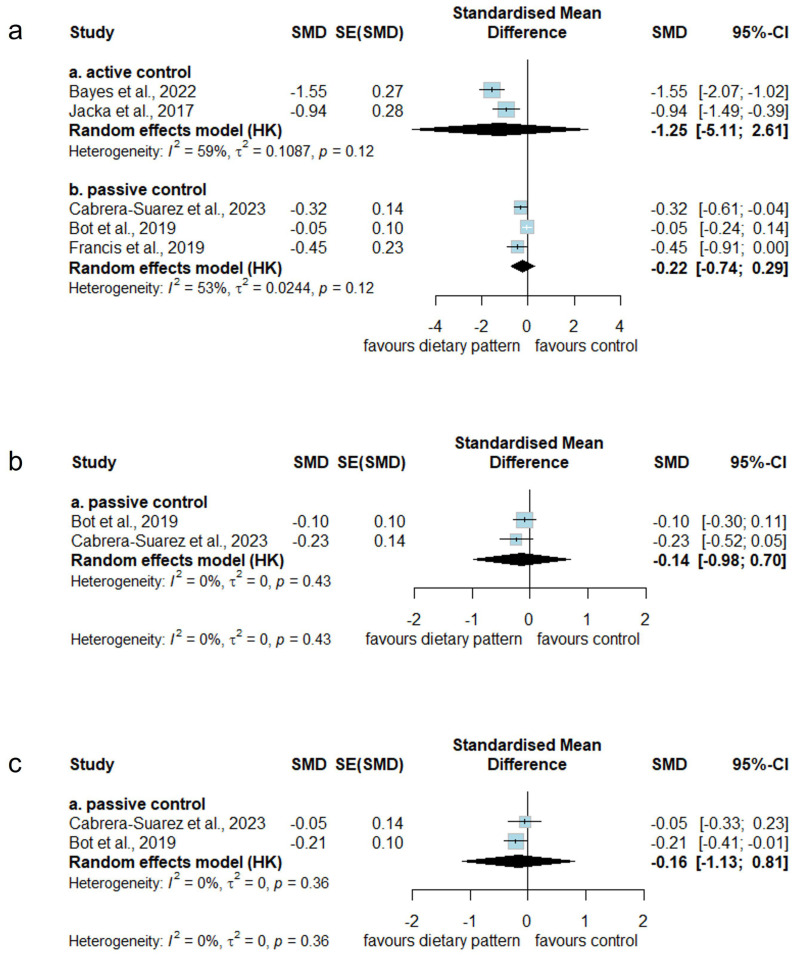
Effect of Mediterranean dietary intervention on depressive symptoms severity compared to active or passive control conditions in (**a**) short, (**b**) intermediate, and (**c**) long-term studies. Cabrera-Suarez et al., 2023 [37], Bayes et al., 2022 [36], Jacka et al., 2017 [41], Bot et al., 2019 [39], Francis et al., 2019 [40].

No significant effect in terms of decreasing the severity of depressive symptoms was found in favor of MD interventions in short-term studies when compared to either active (SMD: −1.25 [95% CI: −5.11 to 2.61]) or passive (SMD: −0.22 [95% CI: −0.74 to 0.29]) control conditions, with substantial between-study heterogeneity (*I^2^* = 0.59%, *p* = 0.12 and *I^2^* = 0.53%, *p* = 0.12 respectively) (Figure 3). The certainty of evidence when compared to the active control group was very low due to the very serious risk of bias, extremely serious imprecision because of the extremely wide confidence interval, and a strong association due to the large effect of SMD (Supplementary File, Table S2). The certainty of evidence when compared to the passive control group was low due to the serious risk of bias, serious indirectness because of the passive control condition, and no large effect of SMD (Supplementary File, Table S3).

Furthermore, MD interventions did not show a significant effect in reducing the severity of depressive symptoms in intermediate-term studies when compared to the passive control condition (SMD: −0.14 [95% CI: −0.98 to 0.70]), with low between-study heterogeneity (*I*^2^ = 0%, *p* = 0.43). No studies used an active control condition in this category (Figure 3). The certainty of evidence when compared to the passive control group was very low due to the serious risk of bias, serious indirectness because of the passive control condition, serious imprecision due to the wide confidence interval, and no large effect of SMD (Supplementary File, Table S3).

For long-term effects on the severity of depressive symptoms, we also found no statistically significant differences in favor of MD interventions compared to the passive control condition (SMD: −0.16 [95% CI: −1.13 to 0.81]), with low between-study heterogeneity (*I^2^* = 0%, *p* = 0.36). No studies used an active control condition in this category (Figure 3). The certainty of evidence when compared to the passive control group was very low due to serious risk of bias, serious indirectness from the passive control condition, very serious imprecision because of the very wide confidence interval, and no large effect of SMD (Supplementary File, Table S3).

#### 3.4.2. Effect on Health-Related Quality of Life

**Figure 4:**
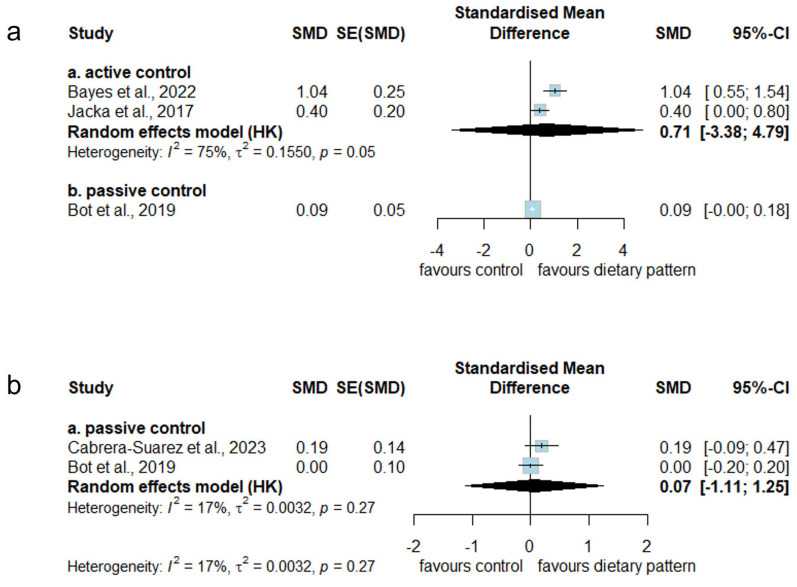
Effect of Mediterranean dietary intervention on health-related quality of life compared to active or passive control conditions in (**a**) short and (**b**) long-term studies. Cabrera-Suarez et al., 2023 [38], Bayes et al., 2022 [36], Jacka et al., 2017 [41], Bot et al., 2019 [39].

Compared to the active control condition, the meta-analysis found no statistically significant short-term effects of MD interventions on health-related quality of life (SMD: 0.71 [95% CI: −3.38 to 4.79]), with considerable between-study heterogeneity (*I^2^* = 75%, *p* = 0.05). For comparisons with the passive control condition, only one measurement was available in this category, which also showed a statistically non-significant effect (Figure 4). The certainty of evidence for comparisons with the active control group was rated as very low due to very serious risk of bias, serious inconsistency arising from different interpretation zones across included studies, extremely serious imprecision due to the extremely wide confidence interval, and lack of a large effect of SMD (Supplementary File, Table S2).

The meta-analysis found no statistically significant long-term effects of MD interventions on health-related quality of life compared to the passive control condition (SMD: 0.07 [95% CI: −1.11 to 1.25]). Low between-study heterogeneity was observed (*I*^2^ = 17%, *p* = 0.27). Notably, no studies in this category employed an active control condition (Figure 4). The certainty of evidence for comparisons with the passive control group was very low due to serious risk of bias, serious indirectness due to the passive control group, very serious imprecision attributed to the very wide confidence interval, and no large effect of SMD (Supplementary File, Table S3).

#### 3.4.3. Safety of Interventions

Two studies reported no adverse events following MD interventions [36,37,38]. One study documented serious adverse events during follow-up, but all were deemed unrelated to the intervention [39]. Two studies did not report safety outcomes [40,41].

#### 3.4.4. Subgroup Analyses, Sensitivity Analyses, and Publication Bias

Subgroup analyses could not be conducted due to an insufficient number of studies in each category. Sensitivity analyses were also not performed, as no significant overall effect of the MD on depressive disorders was observed in any category. Besides, all but one study [41] were assessed as having “some concerns” regarding risk of bias. Since fewer than ten studies were included in each meta-analysis, publication bias was not assessed.

## 4. Discussion

### 4.1. Summary of Evidence

This systematic review and meta-analysis is the first to synthesize the evidence from RCTs examining the effect of different dietary patterns on depressive symptoms severity, remission rate, and health-related quality of life in adult patients with depressive disorders.

All included trials in this systematic review investigated the MD in individuals with major depressive disorder or elevated depression levels. Data from 952 participants (ages 19.6 to 51.1 years, 66.1% women) across five studies were analyzed. The results showed no significant effect of MD interventions on the severity of depressive symptoms or health-related quality of life across all follow-up periods compared to active or passive control groups. Only one study assessed remission rate. Reporting on the safety of the intervention was inconsistent, but the results suggest that the intervention may be considered safe. Between-study heterogeneity ranged from “low” to “considerable”, while most studies had an overall risk of bias rated as “some concerns”. The certainty of evidence for the proposed effects was rated as “very low” for most categories.

### 4.2. Comparison with Previous Systematic Reviews

Previous systematic reviews of RCTs have primarily focused on whole diets or individual foods rather than dietary pattern, as a relatively novel approach to nutritional intervention, and have mostly assessed depression in populations other than patients with diagnosed depressive disorders. Therefore, reviews similar to ours are limited.

The only study [25] that assessed the effect of MD interventions on patients with depression found that the MD significantly reduced depressive symptoms severity among young and middle-aged adults with major depression or mild to moderate depressive symptoms. This review examined the MD both alone, combined with other interventions (such as fish oil supplementation), or as a composite of a lifestyle program (such as performing exercise). Therefore, the results may not fully represent the pure effect of the MD alone on depression. Additionally, the reviewers combined studies with different intervention durations and control conditions when synthesizing the data.

In the review by Swainson et al. [44] on the effect of whole dietary interventions in patients with depressive disorders, five studies were included. However, only two of these studies focused on MD interventions, while the others did not meet the criteria for dietary patterns as defined by the Dietary Guidelines Advisory Committee 2020 [17]. This review did not conduct a meta-analysis but reported general improvements in mood following dietary interventions compared to the control group. In another review [45] on the effects of whole food or diet interventions on depression, the inconsistent nature of the included studies limited the researchers’ ability to synthesize the data. They reported that all studies showed positive outcomes, with depression levels decreasing following dietary interventions. However, only two of the seven included studies met the criteria for dietary patterns, such as MD interventions, being assessed in patients with depression. The remaining included studies focused either on other populations or non-dietary pattern approaches.

There have also been several systematic reviews of RCTs examining the effect of different dietary patterns on depression symptoms in other populations. In the review by Paris et al. [46], which focused on patients with various metabolic conditions (such as metabolic syndrome, type 2 diabetes, hypertension, and fatty liver disease), the authors pooled data from 13 RCTs that assessed overall dietary interventions. They concluded that all dietary interventions significantly improved depression scores. However, only four of these RCTs met the criteria for dietary patterns, specifically focusing on the vegan diet, MD, foods and nutrients for the microbiome, and Mediterranean-DASH diet. On the other hand, combining data from different types of diets to synthesize results is not a well-established practice. The effect of different diets should be analyzed separately and then compared using appropriate statistical methods.

Similar criticisms apply to the study by Firth et al. [47], which assessed the effect of dietary improvements on depression symptoms in a diverse population with various diseases (such as diabetes, hyperlipidemia, knee pain, and breast cancer). The review analyzed data from 16 RCTs and concluded that dietary interventions significantly reduced depressive symptoms. However, only four of the included studies met the criteria for dietary patterns, specifically focusing on the vegan diet and MD. Moreover, considering the diverse population with different diseases—each having unique pathological mechanisms—makes it more challenging to draw a consensus on how a specific dietary pattern affects a particular condition. In another review [48], the effect of short-term (up to 10 days) MD interventions was assessed in healthy participants or individuals with metabolic syndrome. The review reported data from four RCTs without performing a quantitative synthesis and suggested that MD interventions could improve mood in the short term. However, drawing conclusions without pooling the data or performing a meta-analysis limits the interpretability of the findings. For example, in our systematic review, while almost all of the included RCTs showed beneficial effects of the MD on depressive disorders, the final results of the meta-analysis on pooled data were not statistically significant.

There have also been several previous reviews on specific dietary patterns in different populations. In the study by Ghoch et al. [49] on the effect of a ketogenic diet on depression in obese adults, the qualitative analysis demonstrated that low-carbohydrate diets did not have a greater impact on psychosocial outcomes compared to diets with different macronutrient compositions, either in the short term or long term. In another review [50], the effect of a vegetarian/vegan diet on various populations (such as healthy individuals, the obese, and people with diabetes) was assessed. A meta-analysis was conducted on data from three types of studies: RCTs, cross-sectional studies, and cohorts. The results indicated that vegan/vegetarian diets were associated with a higher risk of depression. From a methodological standpoint, pooling data from interventional and observational studies is not appropriate, so these results should be interpreted with caution. Since our study only included RCTs with MD interventions and did not include other dietary patterns, we are unable to compare our results or make any discussions in this regard.

To conclude, discrepancies in the characteristics of participants, interventions, outcomes, and control conditions (PICO components) in the included studies of previous systematic reviews may have contributed to inconsistencies and biases in their findings.

### 4.3. External and Internal Validity

The majority of the included RCTs in our study focused on adults ages 18 years and older, covering a broad spectrum from younger to older adults. Both genders were represented in our study, ensuring that the results are applicable to all genders. Overall, the study samples can be considered representative of the target population, although it should be noted that all the studies were conducted in well-developed countries. Additionally, all the questionnaires used to measure outcomes in the studies were reliable and validated. Furthermore, all studies measured adherence to MD patterns among participants in the intervention group, enhancing the internal validity of the findings.

### 4.4. Strengths and Limitations

Our review has several strengths worth acknowledging. This systematic review employed a search strategy without restrictions on language, publication date, or type of publication. A broad range of literature databases were searched for peer-reviewed journals and gray literature (such as theses and conference abstracts). Additionally, the review considered all types of depressive disorders, not just major depressive disorder, and focused on clinically diagnosed cases of depression rather than self-reported cases. Furthermore, due to the lack of a standardized, comprehensive list of dietary pattern names, we searched all data sources and included all potential dietary patterns in our search strategy, as detailed in the Methods section.

We focused exclusively on single dietary pattern interventions, avoiding combinations with other treatments (e.g., supplementation or lifestyle changes) that could confound the results. To enhance the quality of evidence, we included only RCTs, excluding other trial types. The meta-analysis was conducted separately based on the type of control condition (active or passive), as control group comparability between studies is crucial for pooling data and interpreting meta-analysis results. Lastly, recognizing the significance of follow-up duration in assessing the effects of dietary pattern interventions, we performed separate analyses for short-, intermediate-, and long-term follow-up periods.

There are also some limitations to our review. The primary limitation is the small number of included RCTs, which restricted our ability to perform subgroup analyses and assess publication bias. This issue is particularly important given that half of the studies exhibited “substantial” or “considerable” between-study heterogeneity. Identifying the sources of this heterogeneity would have required subgroup or meta-regression analyses. Additionally, although the results were not statistically significant for any outcome, the overall risk of bias for most studies was categorized as “some concerns”, and the certainty of evidence in most categories was rated as “very low”, potentially affecting the validity of the findings. Furthermore, there were insufficient studies on the effect of dietary patterns on remission rates in patients with depressive disorders, preventing a meta-analysis for this outcome. Moreover, the included RCTs focused solely on MD interventions, limiting our ability to compare their effects with other dietary patterns, which was one of our study objectives. Lastly, it should be considered that nutritional factors may play a more significant role in depressive disorders with a predominance of psychological and environmental influences, rather than those with a genetic background.

### 4.5. Implications for Further Research

This field of research remains underexplored, highlighting the need for more large-scale, high-quality RCTs in the future with active control conditions for evaluating the effects of various dietary patterns on patients with depressive disorders to provide a more robust pool of data for synthesis in upcoming systematic reviews.

## 5. Conclusions

MD interventions appear to have no potential influence on major depressive disorder; however, this finding should be interpreted with caution due to the small number of RCTs available. To draw more definitive conclusions, additional RCTs exploring various dietary patterns and their effects on depressive disorders are needed.

## Figures and Tables

**Table 1:** PICOS inclusion/exclusion criteria of studies.

Parameter	Inclusion Criteria	Exclusion Criteria
Participants	Adults All genders and ethnicities Clinical diagnosis of depressive disorders by physicians or trained health professionals using a recognized diagnostic schedule, by a validated questionnaire, or by report of consumption of a prescribed antidepressant treatment Any type of depressive disorders, including disruptive mood dysregulation disorder, major depressive disorder, persistent depressive disorder (formerly known as dysthymic disorder), premenstrual dysphoric disorder, other specified depressive disorder, unspecified depressive disorder, and unspecified mood disorder, except for the ones in the exclusion criteria No restrictions in terms of sociodemographic characteristics and ongoing pharmacologic or psychologic treatments	Under 18 years of age Pregnant women Self-identified as being depressed without medical diagnosis Some types of depressive disorders, including substance/medication-induced depressive disorder, depressive disorder due to another medical condition (like cancer, dementia, diabetes), and peri-partum/postpartum depression Comorbidity of other psychological or mental disorders like psychosis, bipolar disorders, or schizophrenia
Interventions	All type of dietary patterns, including South Beach, Atkins, ketogenic, gluten free, prudent, Nordic, paleolithic, plant based, Mediterranean, vegetarian, macrobiotic, vegan, Dietary Approaches to Stop Hypertension (DASH), immunonutrition, fermentable oligosaccharides disaccharides monosaccharides and polyols (FODMAP), low sodium, pescetarian, high fiber, whole grain, traditional, polyphenol, anti-oxidant, anti-inflammatory, fruit and vegetable, flexitarian, and fruitarian dietary patterns Single intervention (any provision like dietary advice or instructions, therapy sessions, provision of relevant foods, cooking workshops) without being part of a composite or a combined intervention No limitation on the duration of intervention	Combined (e.g., dietary pattern plus nutrient supplements, exercise) or composite (e.g., dietary pattern as a component of a lifestyle program or a multifaceted program) interventions Diet in terms of intake of single nutrients/bioactive compounds, food items, and food groups Not providing a description of a special dietary pattern, which must include the foods and beverages in the pattern
Controls	Any type of control group, such as no specific intervention (e.g., no treatment, waitlist, usual treatment, habitual diet), functionally inert interventions (e.g., sham or attention control interventions), other dietary patterns, and psychological interventions	n/a
Outcomes	Changes in the following indicators according to standardized rating scales: Depressive symptoms severity Remission/recovery rate Health-related quality of life Safety	Not having any of the intended outcomes
Study design	Randomized controlled trials (RCTs) Published in peer-reviewed journals; gray literature like unpublished data, theses, and conference/meeting papers	Other trials, including uncontrolled trials, nonrandomized controlled trials, before/after studies Other studies, including cohort, case-control, cross-sectional studies, narrative reviews, systematic reviews, meta-analyses, case reports, case series, animal studies

PICOS: population, intervention, controls, outcomes, study design.

**Table 2:** Main characteristics of the included studies.

Reference	Study Characteristics					Participant Characteristics						
	Country	RCT Design	Recruitment Setting			Sample Size (Number)	Completion Rates (Number, %)	Gender (Women, Number, %)		Age (Year, Mean ± SD)	BMI (kg/m^2^)	Type of Disease
Bayes et al. study, 2022 [36]	Australia	Parallel-group, open label	Campaigns with flyers/posters, social media advertisements, databases from previous studies, a recruitment company			T = 75 I = 38 C = 37	T = 72/75 = 96.0% I = 36/38 = 94.7% C = 36/37 = 97.3%	0%		18–25 years T = 22 I = 21.5 ± 2.9 C = 22.5 ± 2.5	n.a.	Major depressive disorder
Cabrera-Su’arez et al. study, 2023 [37,38]	Spain	Multicenter, parallel-group, blinded outcome assessors	In a clinical center			T = 196 I = 103 C = 93	After 1 year: T = 182/196 = 92.8% I = 95/103 = 92.2% C = 87/93 = 93.5% After 2 years: T = 179/196 = 91.3% I = 94/103 = 91.3% C = 85/93 = 91.4%	T = 144/196 = 73.5% I = 74/103 = 71.8% C = 70/93 = 75.3%		18–86 years T = 51.1 ± 14.2 I = 51.16 ± 13.8 C = 51.48 ± 14.7	I = 26.09 ± 4.78 C = 26.04 ± 5.29	Previous major depressive disorder
Jacka et al. study, 2017 [41]	Australia	Parallel-group, blinded outcome assessors, and statistical analyzers	Community-based: flyers in medical waiting rooms, pharmacies, and university campuses; newsletters; potential referral sources (e.g., general practitioners, private psychiatrists, and local psychiatric inpatient units); media interviews; advertisements on social media			T = 67 I = 33 C = 34	T = 56/67 = 83.6% I = 31/33 = 93.9% C = 25/34 = 73.5%	T = 48/67 = 71.6% I = 27/33 = 81.8% C = 21/34 = 61.8%		≥18 years T = 40.3 ± 13.1 I = 37.5 ± 10.7 C = 43.1 ± 14.6	T = 29.5 ± 8 I = 30 ± 9.3 C = 29 ± 6.5	Major depressive disorder
Bot et al. study, 2019 [39]	Germany, Spain, United Kingdom, and the Netherlands	Multicenter, 2 × 2 factorial design, blinded outcome assessors and statistical analyzers	Websites, local advertisements in social media and newspapers, mailings to registered subjects in the general practice setting or in other registers (e.g., municipality registers), posters in public areas, press releases, and via other studies conducted at the four sites			T = 513 I = 256 C = 257	T = 394/513 = 76.8% I = 198/256 = 77.3% C = 196/257 = 76.3%	T = 373/513 = 72.7% I = 193/256 = 75.4% C = 180/257 = 70%		18–75 years I = 46.1 ± 12.8 C = 45.7 ± 13.2	I = 31.2 ± 3.9 C = 31.4 ± 4.1	Elevated depressive symptoms
Francis et al. study, 2019 [40]	Australia	Parallel-group, blinded outcome assessors	Students in undergraduate psychology course, advertisement on the university campus			T = 101 I = 51 C = 50	T = 78/86 = 90.7% I = 39/44 = 88.6% C = 39/42 = 92.8%	T = 48/76 = 63.15% I = 24/38 = 63.15% C = 24/38 = 63.15%		17–35 years I = 19.53 ± 2.05 C = 19.67 ± 2.8	I = 22.07 ± 2.99 C = 22.39 ± 3.37	Elevated depressive symptoms
**References**	**Participants Characteristics**				**Intervention Characteristics**							
	**Severity of Disease**		**Diagnosis Method**	**Concomitant Psychological Treatment**	**Type of Dietary Pattern**	**Duration (Intervention/Follow-Up)**	**Description of the Intervention**			**Measure Time Points**		**Adherence to the Dietary Pattern**
Bayes et al. study, 2022 [36]	Moderate to severe (BDI ≥ 20)		General medical practitioner, BDI-II based on DSM-V	Antidepressant medications, psychotherapy, phone apps focused on mental health	Mediterranean diet	12 weeks, no follow-up	A 60-min appointment by a nutritionist at baseline giving personalized dietary advice, motivational interviewing, and mindful eating. A 60-min follow-up appointment at weeks 6 and 12. The MD based on guidelines of Greece and Spain. Providing a booklet containing information on serving sizes, sample meal plans, compliance checklists. A food hamper for Mediterranean foods. Online personal appointments.			Baseline 6 weeks 12 weeks		MEDAS at baseline, week 6, and week 12
Cabrera-Su’arez et al. study, 2023 [37,38]	Who had suffered at least one depression episode within the last five years and who were in a stage of total or partial clinical remission in the last six months		BDI, SCID, psychiatrists based on DSM-V	Usual clinical care like antidepressants use	Mediterranean diet	2 years	Remote nutritional intervention (changes in diet, information on key Mediterranean foods, menus and specific recipes, answering questions) by registered dietitians by phone and on the internet. Received olive oil as a food hamper.			For HrQoL: baseline 1 year 2 years For depressive symptom severity: Baseline 4 months 8 months 16 months 20 months 24 months		MEDAS at baseline and yearly in the control group and every three months in the intervention group
Jacka et al. study, 2017 [41]	Moderate to severe (MADRS ≥ 18)		MADRS, psychiatry based on DSM-IV	Antidepressant medications, psychotherapy	Mediterranean diet	12 weeks, no follow-up	Seven 60 min sessions of personalized dietary advice, motivational interviewing, mindful eating from a clinical dietician. The first four sessions occurred weekly, and the remaining three sessions occurred every 2 weeks. MD based on guidelines from Greece. A food hamper for MD and recipes and meal plans.			Baseline 12 weeks		ModiMed-Diet score at baseline and week 12
Bot et al. study, 2019 [39]	Mild depressive symptoms (PHQ-9 scores ≥5) and no MDD episode in the past 6 months (MINI5.0)		PHQ-9, MINI5.0	No use of antidepressant drugs or psychological interventions	Mediterranean diet	1 year	Food-related behavioral activation approaches, including self-monitoring, functional analysis, and activity scheduling to improve mood by changing dietary habits and mindful eating. Up to 21 individual sessions meeting at first weekly and then every two weeks, followed by 6 group-based sessions occurring monthly and then bimonthly. Face-to-face sessions. Placebo: 2 pills per day.			Baseline 3 months 6 months 12 months		MooDFOOD diet quality score at baseline and month 12
Francis et al. study, 2019 [40]	Moderate or higher depression symptoms (DASS-21-D ≥ 7)		DASS-21-D	Antidepressant medication or psychological therapy	Mediterranean diet	3 weeks/follow-up for 3 months	Diet intervention instructions from dietician via a video. Sample meal plan and recipes, suggestion for food preparation. Hamper of food items (olive oil, natural nut butter, nuts and seeds, spices). Phone call on Day 7 and 14 to follow up.			Baseline 3 weeks 3 months		Diet Compliance Score questionnaire developed for the study, spectrophotometry
**References**	**Control Characteristics**				**Outcome Characteristics**							**Other Main Consideration**
	**Type of Control Group**		**Description of the Control**		**Depressive Symptom Severity**		**Remission Rates**		**HrQoL**	**Type of Assessment**		
Bayes et al. study, 2022 [36]	Befriending support sessions		The same visit schedule and duration as the diet intervention group. Discussions about neutral topics of interest such as movies, sports, and hobbies. Provided gift card,		BDI-II 21-item		n.a.		WHOQOL-BREF	Self-reported		AMMEND study
Cabrera-Su’arez et al. study, 2023 [37,38]	Usual care		Usual clinical care		BDI questionnaires by phone or through the webpage		n.a.		SF-36 questionnaires by phone or through the webpage	Self-reported		PREDI-DEP study
Jacka et al. study, 2017 [41]	Befriending support sessions		The same visit schedule and length as the diet intervention group. Discussions about neutral topics of interest such as news, sports, and music. Activities such as cards or board games. Provided movie tickets as gift.		MADRS, HADS, POMS, CGI-I		MADRS		WHO wellbeing scale 5	Interview and self-reported		SMILES study
Bot et al. study, 2019 [39]	No active control for diet		Received placebo: 2 pills per day		PHQ-9, IDS30-SR		n.a.		EQ-5D-5L	Self-reported		MooDFOOD study
Francis et al. study, 2019 [40]	Habitual diet		n.a.		CESD-R, DASS-21-D, POMS-A		n.a.		n.a.	Self-reported		Diet, mood, and cognition study

RCT, randomized controlled trial; BMI, body mass index; MEDAS, Mediterranean Diet Adherence Screener; BDI, Beck Depression Inventory; DSM, Diagnostic and Statistical Manual of Mental Disorders; SCID, Structured Clinical Interview for DSM; HrQoL, health-related quality of life; MADRS, Montgomery–Asberg Depression Rating Scale; PHQ, Patient Health Questionnaire; MDD, major depressive disorder; MINI, Mini-International Neuropsychiatric Interview; DASS, Depression, Anxiety, and Stress Scale; WHOQOL, World Health Organization Quality of Life; SF, Short-Form Health Survey; HADS, Hospital Anxiety and Depression Scale; POMS, Profile of Mood States, CGI, Clinical Global Impression; WHO, World Health Organization, IDS, Inventory of Depressive Symptomatology; EQ, EuroQol instrument; CESD, Center for Epidemiologic Studies Depression Scale; MD, Mediterranean diet; SD, standard deviation; T, total; I, intervention group; C, control group.

## Data Availability

The authors declare that the data supporting the findings of this study are available within the paper. Should any raw data files be needed in another format, they will be available from the corresponding author (R.T.) upon reasonable request.

## Supplementary Materials

The following supporting information can be downloaded at: https://www.mdpi.com/article/10.3390/nu17030563/s1, Table S1: Search strategy and syntax for used databases; Table S2: Certainty of evidence compared to active control group; Table S3: Certainty of evidence compared to passive control group.
