# Supplementary material for: Effect of Different Dietary Patterns on Patients with Depressive Disorders: A Systematic Review and Meta-Analysis of Randomized Controlled Trials
Source: Nutrients. 2025 Jan 31;17(3):563. doi: 10.3390/nu17030563 (PMC11820241; doi:10.3390/nu17030563)
Supplement: Supplementary file 1 [file nutrients-17-00563-s001.zip › supplementary/table S1.pdf]

**Search date:**	27/05/2024
**Syntax for PubMed/MEDLINE:**	(“Dietary patterns”[Mesh] OR “Diet* pattern”[tiab] OR “Food pattern”[tiab] OR “Nutrition pattern”[tiab] OR “Eating pattern”[tiab] OR “Food consumption”[tiab] OR “Diet* habit”[tiab] OR “Food habit”[tiab] OR “Nutrition habit”[tiab] OR “Eating habit”[tiab] OR “Diet* quality”[tiab] OR “Nutrition quality”[tiab] OR “Eating quality”[tiab] OR “Diet* Behavior”[tiab] OR “Food Behavior”[tiab] OR “Nutrition Behavior”[tiab] OR “Eating Behavior”[tiab] OR “Diet* profile”[tiab] OR “Food profile”[tiab] OR “Nutrition profile”[tiab] OR “Eating profile”[tiab] OR “Diet* intake”[tiab] OR “Food intake”[tiab] OR “Nutrition intake”[tiab] OR “Diet* style”[tiab] OR “Food style”[tiab] OR “Nutrition style”[tiab] OR “Eating style”[tiab] OR “South Beach Diet*”[tiab] OR “Atkins Diet*”[tiab] OR “Diet, Ketogenic”[Mesh] OR “Ketogenic Diet*”[tiab] OR "Diet, Gluten-Free"[Mesh] OR “Gluten Free diet*”[tiab] OR “prudent diet*”[tiab] OR “Nordic Diet*”[tiab] OR "Diet, Paleolithic"[Mesh] OR “Paleolithic Diet*”[tiab] OR “Paleo Diet*”[tiab] OR “Diet, Plant-Based”[Mesh] OR “plant based diet*”[tiab] OR "Diet, Mediterranean"[Mesh] OR “Mediterranean Diet*”[tiab] OR "Diet, Vegetarian"[Mesh] OR “vegetarian diet*”[tiab] OR Vegetarianism[tiab] OR “Diet, Macrobiotic”[Mesh] OR “Macrobiotic diet*”[tiab] OR "Diet, vegan"[Mesh] OR “vegan diet*”[tiab] OR Veganism[tiab] OR "Dietary Approaches To Stop Hypertension"[Mesh] OR “Dietary Approaches To Stop Hypertension Diet*”[tiab] OR “DASH diet*”[tiab] OR “Immunonutrition Diet”[Mesh] OR “Immunonutrition Diet*”[tiab] OR “FODMAP Diet”[Mesh] OR “FODMAP Diet*”[tiab] OR "Diet, Sodium-Restricted"[Mesh] OR “low sodium diet*”[tiab] OR pescetarian[tiab] OR “high fiber diet*”[tiab] OR “whole-grain diet*”[tiab] OR “traditional diet*”[tiab] OR “Polyphenol Diet*”[tiab] OR “anti-oxidant diet*”[tiab] OR “anti-inflammatory diet*”[tiab] OR “flexitarian diet*”[tiab] OR “fruitarian diet*”[tiab] OR “fruit and vegetable diet*”[tiab])<br>AND<br>(depression[MeSH] OR depression[tiab] OR “Depressive Symptom”[tiab] OR “Depressive Disorder”[MeSH] OR “Depressive Disorder”[tiab] OR “Depressive

	Syndrome"[tiab] OR Melancholia[tiab] OR "Unipolar Depression"[tiab] OR "Depressive Disorder, Major"[MeSH] OR "Major Depressive Disorder"[tiab] OR Paraphrenia[tiab] OR "Dysthymic Disorder"[MeSH] OR "Dysthymic Disorder"[tiab] OR Dysthymia[tiab] OR "Persistent Depressive Disorder"[tiab] OR "Seasonal Affective Disorder"[MeSH] OR "Seasonal Affective Disorder"[tiab] OR "Seasonal Mood Disorder"[tiab] OR "premenstrual Dysphoric disorder"[tiab] OR "Mental Disorders"[MeSH] OR "Mental Disorder"[tiab] OR "Psychiatric Illness"[tiab] OR "Psychiatric Disease"[tiab] OR "Mental Illness"[tiab] OR "Psychiatric Disorder"[tiab] OR "Mood Disorders"[MeSH] OR "Mood Disorder"[tiab] OR "Affective Disorder"[tiab] OR "Affective symptom"[tiab] OR "psychological distress"[MeSH] OR "psychological distress"[tiab] OR "psychological disease"[tiab])<br>AND<br>("randomized controlled trial"[pt] OR "controlled clinical trial"[pt] OR randomized[tiab] OR randomly[tiab] OR trial[tiab] OR groups[tiab] NOT (animals[mh] NOT humans[mh]))
**Syntax for Cochrane CENTRAL:**	([mh "Dietary patterns"] OR "Diet pattern":ti,ab OR "Food pattern":ti,ab OR "Nutrition pattern":ti,ab OR "Eating pattern":ti,ab OR "Food consumption":ti,ab OR "Diet habit":ti,ab OR "Food habit":ti,ab OR "Nutrition habit":ti,ab OR "Eating habit":ti,ab OR "Diet quality":ti,ab OR "Nutrition quality":ti,ab OR "Eating quality":ti,ab OR "Diet Behavior":ti,ab OR "Food Behavior":ti,ab OR "Nutrition Behavior":ti,ab OR "Eating Behavior":ti,ab OR "Diet profile":ti,ab OR "Food profile":ti,ab OR "Nutrition profile":ti,ab OR "Eating profile":ti,ab OR "Diet intake":ti,ab OR "Food intake":ti,ab OR "Nutrition intake":ti,ab OR "Diet style":ti,ab OR "Food style":ti,ab OR "Nutrition style":ti,ab OR "Eating style":ti,ab OR "South Beach Diet":ti,ab OR "Atkins Diet":ti,ab OR [mh "Diet, Ketogenic"] OR "Ketogenic Diet":ti,ab OR [mh "Diet, Gluten-Free"] OR "Gluten Free diet":ti,ab OR "prudent diet":ti,ab OR "Nordic Diet":ti,ab OR [mh "Diet, Paleolithic"] OR "Paleolithic Diet":ti,ab OR "Paleo Diet":ti,ab OR [mh "Diet, Plant-Based"] OR "plant based diet":ti,ab OR [mh "Diet, Mediterranean"] OR "Mediterranean

	Diet”:ti,ab OR [mh "Diet, Vegetarian"] OR “vegetarian diet”:ti,ab OR Vegetarianism:ti,ab OR [mh “Diet, Macrobiotic”] OR “Macrobiotic diet”:ti,ab OR [mh "Diet, vegan"] OR “vegan diet”:ti,ab OR Veganism:ti,ab OR [mh "Dietary Approaches To Stop Hypertension"] OR “Dietary Approaches To Stop Hypertension Diet”:ti,ab OR “DASH diet”:ti,ab OR [mh “Immunonutrition Diet”] OR “Immunonutrition Diet”:ti,ab OR [mh “FODMAP Diet”] OR “FODMAP Diet”:ti,ab OR [mh "Diet, Sodium-Restricted"] OR “low sodium diet”:ti,ab OR pescetarian:ti,ab OR “high fiber diet”:ti,ab OR “whole-grain diet”:ti,ab OR “traditional diet”:ti,ab OR “Polyphenol Diet”:ti,ab OR “anti-oxidant diet”:ti,ab OR “anti-inflammatory diet”:ti,ab OR “flexitarian diet”:ti,ab OR “fruitarian diet”:ti,ab OR “fruit and vegetable diet”:ti,ab)<br>AND<br>([mh depression] OR depression:ti,ab OR “Depressive Symptom”:ti,ab OR [mh “Depressive Disorder”] OR “Depressive Disorder”:ti,ab OR “Depressive Syndrome”:ti,ab OR Melancholia:ti,ab OR “Unipolar Depression”:ti,ab OR [mh “Depressive Disorder, Major”] OR “Major Depressive Disorder”:ti,ab OR Paraphrenia:ti,ab OR [mh “Dysthymic Disorder”] OR “Dysthymic Disorder”:ti,ab OR Dysthymia:ti,ab OR “Persistent Depressive Disorder”:ti,ab OR [mh “Seasonal Affective Disorder”] OR “Seasonal Affective Disorder”:ti,ab OR “Seasonal Mood Disorder”:ti,ab OR “premenstrual Dysphoric disorder”:ti,ab OR [mh “Mental Disorders”] OR “Mental Disorder”:ti,ab OR “Psychiatric Illness”:ti,ab OR “Psychiatric Disease”:ti,ab OR “Mental Illness”:ti,ab OR “Psychiatric Disorder”:ti,ab OR [mh “Mood Disorders”] OR “Mood Disorder”:ti,ab OR “Affective Disorder”:ti,ab OR “Affective symptom”:ti,ab OR [mh “psychological distress”] OR “psychological distress”:ti,ab OR “psychological disease”:ti,ab)
**Syntax for Embase:**	(‘Diet* pattern’:ti,ab OR ‘Food pattern’:ti,ab OR ‘Nutrition pattern’:ti,ab OR ‘Eating pattern’:ti,ab OR ‘Food consumption’:ti,ab OR ‘Diet* habit’:ti,ab OR ‘Food habit’:ti,ab OR ‘Nutrition habit’:ti,ab OR ‘Eating habit’:ti,ab OR ‘Diet* quality’:ti,ab OR ‘Nutrition quality’:ti,ab OR ‘Eating quality’:ti,ab OR ‘Diet* Behavior’:ti,ab OR ‘Food Behavior’:ti,ab OR ‘Nutrition Behavior’:ti,ab OR ‘Eating

	Behavior’:ti,ab OR ‘Diet* profile’:ti,ab OR ‘Food profile’:ti,ab OR ‘Nutrition profile’:ti,ab OR ‘Eating profile’:ti,ab OR ‘Diet* intake’:ti,ab OR ‘Food intake’:ti,ab OR ‘Nutrition intake’:ti,ab OR ‘Diet* style’:ti,ab OR ‘Food style’:ti,ab OR ‘Nutrition style’:ti,ab OR ‘Eating style’:ti,ab OR ‘South Beach Diet*’:ti,ab OR ‘Atkins Diet*’:ti,ab OR ‘Ketogenic Diet*’:ti,ab OR ‘Gluten Free diet*’:ti,ab OR ‘prudent diet*’:ti,ab OR ‘Nordic Diet*’:ti,ab OR ‘Paleolithic Diet*’:ti,ab OR ‘Paleo Diet*’:ti,ab OR ‘plant based diet*’:ti,ab OR ‘Mediterranean Diet*’:ti,ab OR ‘vegetarian diet*’:ti,ab OR ‘Vegetarianism’:ti,ab OR ‘Macrobiotic diet*’:ti,ab OR ‘vegan diet*’:ti,ab OR ‘Veganism’:ti,ab OR ‘Dietary Approaches To Stop Hypertension Diet*’:ti,ab OR ‘DASH diet*’:ti,ab OR ‘Immunonutrition Diet*’:ti,ab OR ‘FODMAP Diet*’:ti,ab OR ‘low sodium diet*’:ti,ab OR ‘pescetarian’:ti,ab OR ‘high fiber diet*’:ti,ab OR ‘whole-grain diet*’:ti,ab OR ‘traditional diet*’:ti,ab OR ‘Polyphenol Diet*’:ti,ab OR ‘anti-oxidant diet*’:ti,ab OR ‘anti-inflammatory diet*’:ti,ab OR ‘flexitarian diet*’:ti,ab OR ‘fruitarian diet*’:ti,ab OR ‘fruit and vegetable diet*’:ti,ab)<br>AND<br>(‘Depression’:ti,ab OR ‘Depressive Symptom’:ti,ab OR ‘Depressive Disorder’:ti,ab OR ‘Depressive Syndrome’:ti,ab OR ‘Melancholia’:ti,ab OR ‘Unipolar Depression’:ti,ab OR ‘Major Depressive Disorder’:ti,ab OR ‘Paraphrenia’:ti,ab OR ‘Dysthymic Disorder’:ti,ab OR ‘Dysthymia’:ti,ab OR ‘Persistent Depressive Disorder’:ti,ab OR ‘Seasonal Affective Disorder’:ti,ab OR ‘Seasonal Mood Disorder’:ti,ab OR ‘premenstrual Dysphoric disorder’:ti,ab OR ‘Mental Disorder’:ti,ab OR ‘Psychiatric Illness’:ti,ab OR ‘Psychiatric Disease’:ti,ab OR ‘Mental Illness’:ti,ab OR ‘Psychiatric Disorder’:ti,ab OR ‘Mood Disorder’:ti,ab OR ‘Affective Disorder’:ti,ab OR ‘Affective symptom’:ti,ab OR ‘psychological distress’:ti,ab OR ‘psychological disease’:ti,ab)<br>AND<br>((‘randomized controlled trial’/exp OR ‘controlled clinical trial’/de OR random*:ti,ab,tt OR ‘randomization’/de OR ‘intermethod comparison’/de OR placebo:ti,ab,tt OR (compare:ti,tt OR compared:ti,tt OR comparison:ti,tt) OR ((evaluated:ab OR evaluate:ab OR evaluating:ab OR assessed:ab OR assess:ab)

	AND (compare:ab OR compared:ab OR comparing:ab OR comparison:ab)) OR (open NEXT/1 label):ti,ab,tt OR ((double OR single OR doubly OR singly) NEXT/1 (blind OR blinded OR blindly)):ti,ab,tt OR ‘double blind procedure’/de OR (parallel NEXT/1 group*):ti,ab,tt OR (crossover:ti,ab,tt OR ‘cross over’:ti,ab,tt) OR ((assign* OR match OR matched OR allocation) NEAR/6 (alternate OR group OR groups OR intervention OR interventions OR patient OR patients OR subject OR subjects OR participant OR participants)):ti,ab,tt OR (assigned:ti,ab,tt OR allocated:ti,ab,tt) OR (controlled NEAR/8 (study OR design OR trial)):ti,ab,tt OR (volunteer:ti,ab,tt OR volunteers:ti,ab,tt) OR ‘human experiment’/de OR trial:ti,tt) NOT ((((random* NEXT/1 sampl* NEAR/8 (‘cross section*’ OR questionnaire* OR survey OR surveys OR database or databases)):ti,ab,tt) NOT (‘comparative study’/de OR ‘controlled study’/de OR ‘randomised controlled’:ti,ab,tt OR ‘randomized controlled’:ti,ab,tt OR ‘randomly assigned’:ti,ab,tt)) OR (‘cross-sectional study’/de NOT (‘randomized controlled trial’/exp OR ‘controlled clinical study’/de OR ‘controlled study’/de OR ‘randomised controlled’:ti,ab,tt OR ‘randomized controlled’:ti,ab,tt OR ‘control group’:ti,ab,tt OR ‘control groups’:ti,ab,tt)) OR (‘case control*’:ti,ab,tt AND random*:ti,ab,tt NOT (‘randomised controlled’:ti,ab,tt OR ‘randomized controlled’:ti,ab,tt)) OR (‘systematic review’:ti,tt NOT (trial:ti,tt OR study:ti,tt)) OR (nonrandom*:ti,ab,tt NOT random*:ti,ab,tt) OR ‘random field*’:ti,ab,tt OR (‘random cluster’ NEAR/4 sampl*):ti,ab,tt OR (review:ab AND review:it) NOT trial:ti,tt OR (‘we searched’:ab AND (review:ti,tt OR review:it)) OR ‘update review’:ab OR (databases NEAR/5 searched):ab OR ((rat:ti,tt OR rats:ti,tt OR mouse:ti,tt OR mice:ti,tt OR swine:ti,tt OR porcine:ti,tt OR murine:ti,tt OR sheep:ti,tt OR lambs:ti,tt OR pigs:ti,tt OR piglets:ti,tt OR rabbit:ti,tt OR rabbits:ti,tt OR cat:ti,tt OR cats:ti,tt OR dog:ti,tt OR dogs:ti,tt OR cattle:ti,tt OR bovine:ti,tt OR monkey:ti,tt OR monkeys:ti,tt OR trout:ti,tt OR marmoset*:ti,tt) AND ‘animal experiment’/de) OR (‘animal experiment’/de NOT (‘human experiment’/de OR ‘human’/de))))
**Syntax for PsycINFO via EBSCO:**	(“Diet* pattern” OR “Food pattern” OR “Nutrition pattern” OR “Eating pattern” OR “Food consumption” OR “Diet* habit” OR “Food habit” OR “Nutrition habit” OR

	"Eating habit" OR "Diet* quality" OR "Nutrition quality" OR "Eating quality" OR "Diet* Behavior" OR "Food Behavior" OR "Nutrition Behavior" OR "Eating Behavior" OR "Diet* profile" OR "Food profile" OR "Nutrition profile" OR "Eating profile" OR "Diet* intake" OR "Food intake" OR "Nutrition intake" OR "Diet* style" OR "Food style" OR "Nutrition style" OR "Eating style" OR "South Beach Diet*" OR "Atkins Diet*" OR "Ketogenic Diet*" OR "Gluten Free diet*" OR "prudent diet*" OR "Nordic Diet*" OR "Paleolithic Diet*" OR "Paleo Diet*" OR "plant based diet*" OR "Mediterranean Diet*" OR "vegetarian diet*" OR Vegetarianism OR "Macrobiotic diet*" OR "vegan diet*" OR Veganism OR "Dietary Approaches To Stop Hypertension Diet*" OR "DASH diet*" OR "Immunonutrition Diet*" OR "FODMAP Diet*" OR "low sodium diet*" OR pescetarian OR "high fiber diet*" OR "whole-grain diet*" OR "traditional diet*" OR "Polyphenol Diet*" OR "anti-oxidant diet*" OR "anti-inflammatory diet*" OR "flexitarian diet*" OR "fruitarian diet*" OR "fruit and vegetable diet*")<br>AND<br>(depression OR "Depressive Symptom" OR "Depressive Disorder" OR "Depressive Syndrome" OR Melancholia OR "Unipolar Depression" OR "Major Depressive Disorder" OR Paraphrenia OR "Dysthymic Disorder" OR Dysthymia OR "Persistent Depressive Disorder" OR "Seasonal Affective Disorder" OR "Seasonal Mood Disorder" OR "premenstrual Dysphoric disorder" OR "Mental Disorder" OR "Psychiatric Illness" OR "Psychiatric Disease" OR "Mental Illness" OR "Psychiatric Disorder" OR "Mood Disorder" OR "Affective Disorder" OR "Affective symptom" OR "psychological distress" OR "psychological disease")
**Syntax for Scopus:**	(TITLE-ABS("Diet* pattern") OR TITLE-ABS("Food pattern") OR TITLE-ABS("Nutrition pattern") OR TITLE-ABS("Eating pattern") OR TITLE-ABS("Food consumption") OR TITLE-ABS("Diet* habit") OR TITLE-ABS("Food habit") OR TITLE-ABS("Nutrition habit") OR TITLE-ABS("Eating habit") OR TITLE-ABS("Diet* quality") OR TITLE-ABS("Nutrition quality") OR TITLE-ABS("Eating quality") OR TITLE-ABS("Diet* Behavior") OR TITLE-ABS("Food Behavior") OR TITLE-ABS("Nutrition Behavior") OR TITLE-ABS("Eating

	Behavior”) OR TITLE-ABS(“Diet* profile”) OR TITLE-ABS(“Food profile”) OR TITLE-ABS(“Nutrition profile”) OR TITLE-ABS(“Eating profile”) OR TITLE-ABS(“Diet* intake”) OR TITLE-ABS(“Food intake”) OR TITLE-ABS(“Nutrition intake”) OR TITLE-ABS(“Diet* style”) OR TITLE-ABS(“Food style”) OR TITLE-ABS(“Nutrition style”) OR TITLE-ABS(“Eating style”) OR TITLE-ABS(“South Beach Diet*”) OR TITLE-ABS(“Atkins Diet*”) OR TITLE-ABS(“Ketogenic Diet*”) OR TITLE-ABS(“Gluten Free diet*”) OR TITLE-ABS(“prudent diet*”) OR TITLE-ABS(“Nordic Diet*”) OR TITLE-ABS(“Paleolithic Diet*”) OR TITLE-ABS(“Paleo Diet*”) OR TITLE-ABS(“plant based diet*”) OR TITLE-ABS(“Mediterranean Diet*”) OR TITLE-ABS(“vegetarian diet*”) OR TITLE-ABS(Vegetarianism) OR TITLE-ABS(“Macrobiotic diet*”) OR TITLE-ABS(“vegan diet*”) OR TITLE-ABS(Veganism) OR TITLE-ABS(“Dietary Approaches To Stop Hypertension Diet*”) OR TITLE-ABS(“DASH diet*”) OR TITLE-ABS(“Immunonutrition Diet*”) OR TITLE-ABS(“FODMAP Diet*”) OR TITLE-ABS(“low sodium diet*”) OR TITLE-ABS(pescetarian) OR TITLE-ABS(“high fiber diet*”) OR TITLE-ABS(“whole-grain diet*”) OR TITLE-ABS(“traditional diet*”) OR TITLE-ABS(“Polyphenol Diet*”) OR TITLE-ABS(“anti-oxidant diet*”) OR TITLE-ABS(“anti-inflammatory diet*”) OR TITLE-ABS(“flexitarian diet*”) OR TITLE-ABS(“fruitarian diet*”) OR TITLE-ABS(“fruit and vegetable diet*”))<br>AND<br>(TITLE-ABS(depression) OR TITLE-ABS(“Depressive Symptom”) OR TITLE-ABS(“Depressive Disorder”) OR TITLE-ABS(“Depressive Syndrome”) OR TITLE-ABS(Melancholia) OR TITLE-ABS(“Unipolar Depression”) OR TITLE-ABS(“Major Depressive Disorder”) OR TITLE-ABS(Paraphrenia) OR TITLE-ABS(“Dysthymic Disorder”) OR TITLE-ABS(Dysthymia) OR TITLE-ABS(“Persistent Depressive Disorder”) OR TITLE-ABS(“Seasonal Affective Disorder”) OR TITLE-ABS(“Seasonal Mood Disorder”) OR TITLE-ABS(“premenstrual Dysphoric disorder”) OR TITLE-ABS(“Mental Disorder”) OR TITLE-ABS(“Psychiatric Illness”) OR TITLE-ABS(“Psychiatric Disease”) OR TITLE-ABS(“Mental Illness”) OR TITLE-ABS(“Psychiatric Disorder”) OR

	TITLE-ABS("Mood Disorder") OR TITLE-ABS("Affective Disorder") OR TITLE-ABS("Affective symptom") OR TITLE-ABS("psychological distress") OR TITLE-ABS("psychological disease"))
**Syntax for ProQuest**	(AB,TI("Diet* pattern") OR AB,TI("Food pattern") OR AB,TI("Nutrition pattern") OR AB,TI("Eating pattern") OR AB,TI("Food consumption") OR AB,TI("Diet* habit") OR AB,TI("Food habit") OR AB,TI("Nutrition habit") OR AB,TI("Eating habit") OR AB,TI("Diet* quality") OR AB,TI("Nutrition quality") OR AB,TI("Eating quality") OR AB,TI("Diet* Behavior") OR AB,TI("Food Behavior") OR AB,TI("Nutrition Behavior") OR AB,TI("Eating Behavior") OR AB,TI("Diet* profile") OR AB,TI("Food profile") OR AB,TI("Nutrition profile") OR AB,TI("Eating profile") OR AB,TI("Diet* intake") OR AB,TI("Food intake") OR AB,TI("Nutrition intake") OR AB,TI("Diet* style") OR AB,TI("Food style") OR AB,TI("Nutrition style") OR AB,TI("Eating style") OR AB,TI("South Beach Diet*") OR AB,TI("Atkins Diet*") OR AB,TI("Ketogenic Diet*") OR AB,TI("Gluten Free diet*") OR AB,TI("prudent diet*") OR AB,TI("Nordic Diet*") OR AB,TI("Paleolithic Diet*") OR AB,TI("Paleo Diet*") OR AB,TI("plant based diet*") OR AB,TI("Mediterranean Diet*") OR AB,TI("vegetarian diet*") OR AB,TI(Vegetarianism) OR AB,TI("Macrobiotic diet*") OR AB,TI("vegan diet*") OR AB,TI(Veganism) OR AB,TI("Dietary Approaches To Stop Hypertension Diet*") OR AB,TI("DASH diet*") OR AB,TI("Immunonutrition Diet*") OR AB,TI("FODMAP Diet*") OR AB,TI("low sodium diet*") OR AB,TI(pescetarian) OR AB,TI("high fiber diet*") OR AB,TI("whole-grain diet*") OR AB,TI("traditional diet*") OR AB,TI("Polyphenol Diet*") OR AB,TI("anti-oxidant diet*") OR AB,TI("anti-inflammatory diet*") OR AB,TI("flexitarian diet*") OR AB,TI("fruitarian diet*") OR AB,TI("fruit and vegetable diet*"))<br>AND<br>(AB,TI(depression) OR AB,TI("Depressive Symptom") OR AB,TI("Depressive Disorder") OR AB,TI("Depressive Syndrome") OR AB,TI(Melancholia) OR AB,TI("Unipolar Depression") OR AB,TI("Major Depressive Disorder") OR AB,TI(Paraphrenia) OR AB,TI("Dysthymic Disorder") OR AB,TI(Dysthymia) OR

	AB,TI("Persistent Depressive Disorder") OR AB,TI("Seasonal Affective Disorder") OR AB,TI("Seasonal Mood Disorder") OR AB,TI("premenstrual Dysphoric disorder") OR AB,TI("Mental Disorder") OR AB,TI("Psychiatric Illness") OR AB,TI("Psychiatric Disease") OR AB,TI("Mental Illness") OR AB,TI("Psychiatric Disorder") OR AB,TI("Mood Disorder") OR AB,TI("Affective Disorder") OR AB,TI("Affective symptom") OR AB,TI("psychological distress") OR AB,TI("psychological disease"))
