# Supplementary material for: Effect of Different Dietary Patterns on Patients with Depressive Disorders: A Systematic Review and Meta-Analysis of Randomized Controlled Trials
Source: Nutrients. 2025 Jan 31;17(3):563. doi: 10.3390/nu17030563 (PMC11820241; doi:10.3390/nu17030563)
Supplement: Supplementary file 1 [file nutrients-17-00563-s001.zip › supplementary/Table S2.pdf]

Summary of findings:

Dietary pattern compared to active control for depressive disorders

Patient or population: depressive disorders
Setting:
Intervention: dietary pattern
Comparison: active control

Outcomes	Anticipated absolute effects* (95% CI)		Relative effect (95% CI)	№ of participants (studies)	Certainty of the evidence (GRADE)	Comments
	Risk with active control	Risk with dietary pattern				
depressive symptom severity-short term	-	SMD **1.25 SD lower** (5.11 lower to 2.61 higher)	-	128 (2 RCTs)	⨁◯◯◯ Very low[a,b,c]	
health related quality of life-short term	-	SMD **0.71 SD higher** (3.38 lower to 4.79 higher)	-	128 (2 RCTs)	⨁◯◯◯ Very low[a,b,d]	

***The risk in the intervention group** (and its 95% confidence interval) is based on the assumed risk in the comparison group and the **relative effect** of the intervention (and its 95% CI).

CI: confidence interval; **SMD:** standardised mean difference

GRADE Working Group grades of evidence
High certainty: we are very confident that the true effect lies close to that of the estimate of the effect.
Moderate certainty: we are moderately confident in the effect estimate: the true effect is likely to be close to the estimate of the effect, but there is a possibility that it is substantially different.
Low certainty: our confidence in the effect estimate is limited: the true effect may be substantially different from the estimate of the effect.
Very low certainty: we have very little confidence in the effect estimate: the true effect is likely to be substantially different from the estimate of effect.

Explanations

a. At least one study has high risk of bias
b. Confidence interval crosses 3 interpretation zones completely
c. Large effect of SMD
d. The results of the studies are in 2 interpretation zones
