# Supplementary material for: Effect of Different Dietary Patterns on Patients with Depressive Disorders: A Systematic Review and Meta-Analysis of Randomized Controlled Trials
Source: Nutrients. 2025 Jan 31;17(3):563. doi: 10.3390/nu17030563 (PMC11820241; doi:10.3390/nu17030563)
Supplement: Supplementary file 1 [file nutrients-17-00563-s001.zip › supplementary/Table S3.pdf]

Summary of findings:

Dietary pattern compared to passive control for depressive disorders

Patient or population: depressive disorders
Setting:
Intervention: dietary pattern
Comparison: passive control

Outcomes	Anticipated absolute effects* (95% CI)		Relative effect (95% CI)	№ of participants (studies)	Certainty of the evidence (GRADE)	Comments
	**Risk with passive control**	**Risk with dietary pattern**				
depressive symptom severity- short term	-	SMD **0.22 SD lower** (0.74 lower to 0.29 higher)	-	689 (3 RCTs)	⊕⊕◯◯ Low[a,b]	
depressive symptom severity- intermediate term	-	SMD **0.14 SD lower** (0.98 lower to 0.7 higher)	-	580 (2 RCTs)	⊕◯◯◯ Very low[a,b,c]	
depressive symptom severity- long term	-	SMD **0.16 SD lower** (1.13 lower to 0.81 higher)	-	583 (2 RCTs)	⊕◯◯◯ Very low[a,b,d]	
health related quality of life-long term	-	SMD **0.07 SD higher** (1.11 lower to 1.25 higher)	-	583 (2 RCTs)	⊕◯◯◯ Very low[a,b,d]	

***The risk in the intervention group** (and its 95% confidence interval) is based on the assumed risk in the comparison group and the **relative effect** of the intervention (and its 95% CI).

CI: confidence interval; **SMD:** standardised mean difference

GRADE Working Group grades of evidence
High certainty: we are very confident that the true effect lies close to that of the estimate of the effect.
Moderate certainty: we are moderately confident in the effect estimate: the true effect is likely to be close to the estimate of the effect, but there is a possibility that it is substantially different.
Low certainty: our confidence in the effect estimate is limited: the true effect may be substantially different from the estimate of the effect.
Very low certainty: we have very little confidence in the effect estimate: the true effect is likely to be substantially different from the estimate of effect.

Explanations

a. All of the studies have some concerns for risk of bias
b. Interested control group is not provided across the studies
c. Confidence interval crosses 2 interpretation zones
d. Confidence interval crosses near 3 interpretation zones
